# Supplementary material for: High variation in immune responses and parasite phenotypes in naturally acquired Trypanosoma cruzi infection in a captive non-human primate breeding colony in Texas, USA
Source: PLoS Negl Trop Dis. 2021 Mar 31;15(3):e0009141. doi: 10.1371/journal.pntd.0009141 (PMC8041201; doi:10.1371/journal.pntd.0009141)
Supplement: S1 Fig — Male (n = 19) and female (n = 31) macaques in the study had similar ages and displayed comparable minimum length of infection ranging from 1.5 to 8.5 years. (PDF) [file pntd.0009141.s001.pdf]

S1 Fig.

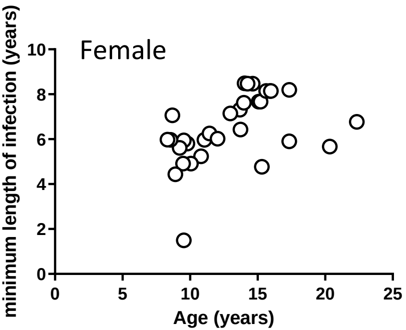

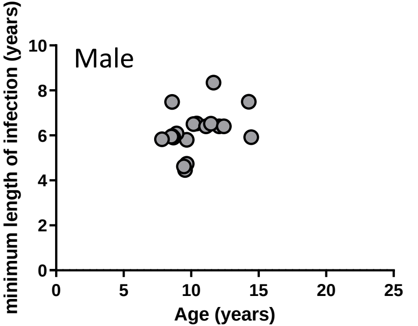

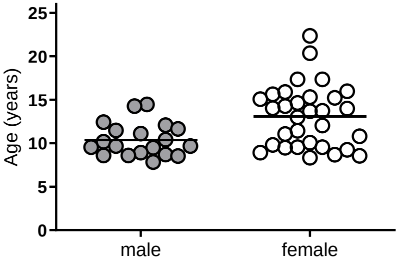

S1 Fig. Macaques in the study had been infected at least for an average duration of 6.5 years. Male (n=19) and female (n=31) macaques in the study had similar ages and displayed comparable minimum length of infection ranging from 1.5 to 8.5 years.
