# Supplementary material for: High variation in immune responses and parasite phenotypes in naturally acquired Trypanosoma cruzi infection in a captive non-human primate breeding colony in Texas, USA
Source: PLoS Negl Trop Dis. 2021 Mar 31;15(3):e0009141. doi: 10.1371/journal.pntd.0009141 (PMC8041201; doi:10.1371/journal.pntd.0009141)
Supplement: S2 Fig — Parasite load in blood detected by qPCR (A) and minimum length of infection (B) in macaques infected with T. cruzi isolates belonging to lineages TcI (n = 33 in A; n = 27 in B) and TcIV (n = 7). ns: non-significant by Mann Whitney test. (PDF) [file pntd.0009141.s002.pdf]

S2 Fig.

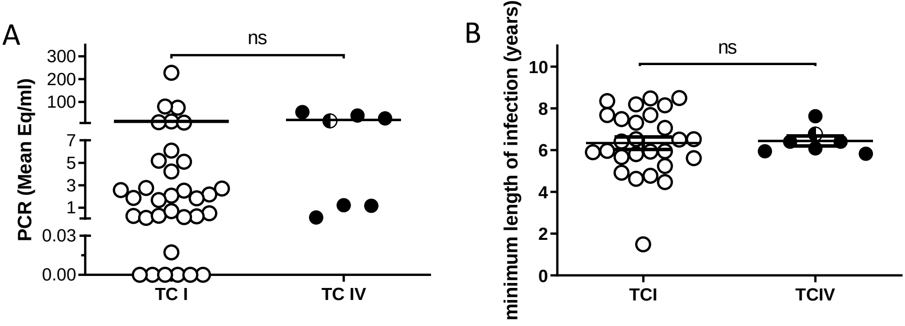

S2 Fig. Parasite levels in blood and minimum length of infection were similar in macaques infected with either TcI or TcIV. Parasite load in blood detected by qPCR (A) and minimum length of infection (B) in macaques infected with *T. cruzi* isolates belonging to lineages TcI (n=33 in A; n=27 in B) and TcIV (n=7). ns: non-significant by Mann Whitney test.
