# Supplementary material for: High variation in immune responses and parasite phenotypes in naturally acquired Trypanosoma cruzi infection in a captive non-human primate breeding colony in Texas, USA
Source: PLoS Negl Trop Dis. 2021 Mar 31;15(3):e0009141. doi: 10.1371/journal.pntd.0009141 (PMC8041201; doi:10.1371/journal.pntd.0009141)
Supplement: S3 Fig — Parasite level in blood determined by qPCR (A) and minimum length of infection (B) in animals displaying no symptoms (no symptom; n = 28), poor general health condition (poor health; n = 9) or cardiac anomalies (cardiac anom; n = 13). ns: non-significant by Mann Whitney test. (PDF) [file pntd.0009141.s003.pdf]

S3 Fig.

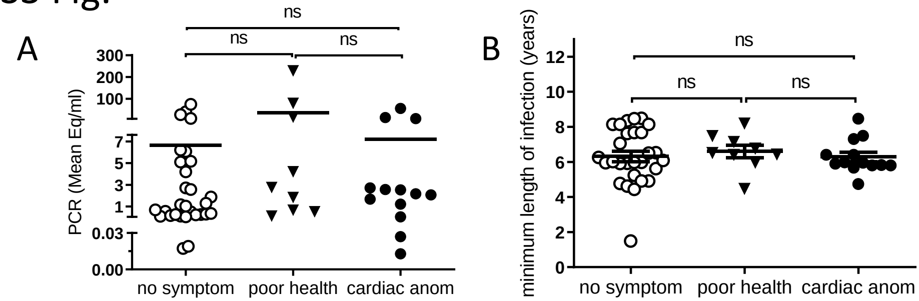

S3 Fig. Health condition of seropositive macaques does not correlate with parasite load or minimum length of infection. Parasite level in blood determined by qPCR (A) and minimum length of infection (B) in animals displaying no symptoms (no symptom; n=28), poor general health condition (poor health; n=9) or cardiac anomalies (cardiac anom; n=13). ns: non-significant by Mann Whitney test.
