# Supplementary material for: High variation in immune responses and parasite phenotypes in naturally acquired Trypanosoma cruzi infection in a captive non-human primate breeding colony in Texas, USA
Source: PLoS Negl Trop Dis. 2021 Mar 31;15(3):e0009141. doi: 10.1371/journal.pntd.0009141 (PMC8041201; doi:10.1371/journal.pntd.0009141)
Supplement: Attachment — Submitted filename: Response to reviewers.docx [file pntd.0009141.s005.docx]

We thank the editors and the reviewers for the rapid and complete review of our submission. We believe we have addressed all of the review comments and summarize these responses and the changes to the manuscript below. Our specific responses are indicated in italics.

Two versions of the manuscript, one with the changed tracked, have also been returned.

Reviewer #1:
- Not all 64 animals were assayed in all techniques through the study. It would facilitate reading if the number of participant animals was indicated in the legends of each graph.

*Done*

- It should be included the information on the Ig isotypes measured in the study, considering that the isotype gives valuable information about the host's immunity branches involved in their anti-cruzi response.

*Done*

- Fig 6A does not show any TcIV results, although in lines 208-209 it is mentioned the authors found TcIV-only samples. The authors should show individual results for all animals in supplementary material.

*Done, see new raw data file*

- The in vivo experiments (Fig 7) did not add much value to the study. The conclusion that "... isolates ... showed a number of treatment failures, similar to what has been reported with other more “benznidazole-resistant” T. cruzi lines [27]" without deeper exploration of the genetics of each isolate andqor the causes for such discrepancies, was not worth the time, resources, and mice invested in these in vitro experiments.

*We strongly disagree with this view. A primary focus to this work is to highlight the phenotypic differences between T. cruzi isolates circulating in the same, highly contained population of hosts. The variability in susceptibility to anti-T. cruzi compounds is one, and indeed among the most important, of these phenotypic differences and supports the view that the well-documented differential response to benznidazole treatment in humans could be in part due to variations in the parasites infecting (even closely related) individuals. The point was not to identify the basis of these differences, just like we have not attempted to identify the genetic basis of differential immune responses, etc., in relationship to the lines infecting these individual macaques. The reviewer may be aware that the genetic basis of such variations (not “discrepancies”) in drug sensitivities has not been identified for any* T. cruzi *strains.*

Thirteen macaques were soropositive but had negative qPCR and culture/qPCR results. These macaques were not evaluated by EKG. But then, on line 321, the authors state that "... health status and infection-related pathology ... correlated with the antibody response against a T. cruzi lysate but not with parasite levels in blood or the other immune parameters measured here." Based in their own findings, i.e. that cardiac anomalies correlated only with antibody response, the authors should have included the EKG results from the 13 macaques that were soropositive but had negative qPCR. These 13 macaques is the group that will prove their point from line 321, and these results were not shown.

*We have included all the EKG/health data in the supplemental raw data file. We have also modified the Discussion on the point of the association of low antibody levels with lower incidence of disease.*

- In fact, only 12 out of 64 macaques had EKG exams performed (line 382). I consider this a relevant flaw in the study, since it claims to correlate clinical findings with immunological and parasitological findings (line 289). Furthermore, EKG excerpts from individual macaques must be included as supplementary materials. As it is now, the manuscript requires the reader to accept third party interpretation of the EKG results and the "poor health" status from Fig 8 without having access to clinical and laboratorial parameters used to define "poor health" or the original EKG readings. In this sense, the people involved in performing and interpreting the clinical data and EKGs should share authorship (and accountability) for the findings, instead of just being mentioned in the acknowledgements section (lines 534-535). If this study is reusing previous clinical data acquired routinely at this macaques facility, this must be made clear in the manuscript.

Full disclosure of results, supplementary material, and authorships are necessary for supporting the conclusions of the manuscript. Clinical and laboratorial parameters used to define the health status of the macaques should be stated and individual data must be shown, possibly as supplementary material. Individual EKG excerpts for all macaques should be included as supplementary materials.

*Done, see new raw data file*

Reviewer #3: This investigation took advantage of a convenient sample that is not readily available in nature for inquisition. The authors made several important immunologic and scientific findings, that cumulatively suggest that host immune response to T cruzi infection is quite varied and complicated. One minor recommendation, would be to include the actual months samples were collected and to provide information as to whether the primates were inside versus outside. It was stated that primates were in different conditions, and one could wonder whether those outside during peak triatomine season have the potential for reinfection, which could influence the results. Clearly this would be hard to confirm, but it should be noted in the limitations.

*Done*
